# Atlantic salmon populations reveal adaptive divergence of immune related genes - a duplicated genome under selection

**DOI:** 10.1186/s12864-016-2867-z

**Published:** 2016-08-11

**Authors:** Erik Kjærner-Semb, Fernando Ayllon, Tomasz Furmanek, Vidar Wennevik, Geir Dahle, Eero Niemelä, Mikhail Ozerov, Juha-Pekka Vähä, Kevin A. Glover, Carl J. Rubin, Anna Wargelius, Rolf B. Edvardsen

**Affiliations:** 1 Institute of Marine Research, Bergen, Norway; 2 Department of Biology, University of Bergen, Bergen, Norway; 3 Natural Resources Institute Finland, Helsinki, Finland; 4 Kevo Subarctic Research Institute, University of Turku, Turku, Finland; 5 Department of Medical Biochemistry and Microbiology, Uppsala University, Uppsala, Sweden; 6 Association for Water and Environment of Western Uusimaa, Uusimaa, Finland

**Keywords:** Whole genome duplication, Adaptation, Aquaculture, Immune system, GWAS, Resequencing, Selective sweep, SNPs, Salmo salar

## Abstract

**Background:**

Populations of Atlantic salmon display highly significant genetic differences with unresolved molecular basis. These differences may result from separate postglacial colonization patterns, diversifying natural selection and adaptation, or a combination. Adaptation could be influenced or even facilitated by the recent whole genome duplication in the salmonid lineage which resulted in a partly tetraploid species with duplicated genes and regions.

**Results:**

In order to elucidate the genes and genomic regions underlying the genetic differences, we conducted a genome wide association study using whole genome resequencing data from eight populations from Northern and Southern Norway. From a total of ~4.5 million sequencing-derived SNPs, more than 10 % showed significant differentiation between populations from these two regions and ten selective sweeps on chromosomes 5, 10, 11, 13–15, 21, 24 and 25 were identified. These comprised 59 genes, of which 15 had one or more differentiated missense mutation. Our analysis showed that most sweeps have paralogous regions in the partially tetraploid genome, each lacking the high number of significant SNPs found in the sweeps. The most significant sweep was found on Chr 25 and carried several missense mutations in the antiviral *mx* genes, suggesting that these populations have experienced differing viral pressures. Interestingly the second most significant sweep, found on Chr 5, contains two genes involved in the NF-KB pathway (*nkap* and *nkrf*), which is also a known pathogen target that controls a large number of processes in animals.

**Conclusion:**

Our results show that natural selection acting on immune related genes has contributed to genetic divergence between salmon populations in Norway. The differences between populations may have been facilitated by the plasticity of the salmon genome. The observed signatures of selection in duplicated genomic regions suggest that the recently duplicated genome has provided raw material for evolutionary adaptation.

**Electronic supplementary material:**

The online version of this article (doi:10.1186/s12864-016-2867-z) contains supplementary material, which is available to authorized users.

## Background

In addition to being one of the most highly prized freshwater fish for recreational fishing, the Atlantic salmon (*Salmo salar* L.) is one of the most economically important aquaculture species worldwide. Its natural distribution is throughout the North Atlantic, ranging from Long Island Sound to Ungava Bay in the west and from Northern Portugal to the Barents Sea in the east [1]. This distribution is the result of postglacial colonization of ecosystems that became available when the glacial ice retreated about 10,000 years ago [2].

Atlantic salmon is characterised by highly significant, hierarchically structured population genetic divergence, with the largest differences observed between the European and North American lineages [3–5]. This divergence is also observed on a regional scale, presumably as a consequence of the colonization process associated with the retreat of the glacier [6, 7]. Moreover, local scale differentiation exists, for example between neighbouring rivers [8–10] and among tributaries within the same river which might be explained by restricted gene flow, genetic drift and adaptation [11–13].

Atlantic salmon exhibit a relatively complex life history that includes spawning and juvenile rearing in freshwater followed by extended ocean migrations to the feeding grounds [14]. As a consequence, salmon go through several distinct transitions that are characterized by changes in behaviour and physiology [15]. They are also able to adapt to varying local conditions throughout their range of environments [16], exemplified by their ability to inhabit rivers with a wide range of temperatures, from Spain to the colder Arctic latitudes [17]. Previous studies have shown differences in temperature and climate to be associated with genetic differences between salmon populations [7, 18], and latitude also seems to be correlated with allele frequencies of markers relevant to immune response in American and European Atlantic salmon populations, possibly due to temperature induced differences in pathogen-driven selection or other environmental factors [19–21].

In the wild, Atlantic salmon are constantly confronted with a range of pathogens, and have consequently developed numerous innate and adaptive immune mechanisms to overcome infectious challenges [22]. Recent studies suggest that the prevalence of parasites and infectious diseases is increasing in wild populations partly due to global warming [23, 24]. Given the commercial relevance of Atlantic salmon and the recent release of a reference genome [25], particular effort should be made to identify genes targeted by natural selection in wild Atlantic salmon populations that ultimately can lead to optimized aquaculture practices. The potential relevance of these findings for the Atlantic salmon farming industry is exemplified by the identification of Infectious Pancreatic Necrosis (IPN) Virus resistance [26] and age at maturity associated genes [27, 28]. A relatively recent whole genome duplication occurred in the salmonid lineage some 80 million years ago [29], resulting in a partly tetraploid genome undergoing rediploidization. Consequently the genome contains many paralogous regions that could provide raw material for evolution as paralogous genes and regions can diversify and acquire new functions [30].

Based upon the analysis of microsatellite and SNP markers, several studies have demonstrated that there are highly significant genetic differences between Atlantic salmon populations located in the north and south of Norway [31–33]. However, the genomic regions and genes behind the differences have not been investigated in detail, and consequently, the potential adaptive significance of this genetic divergence remains elusive.

**Fig. 1:**
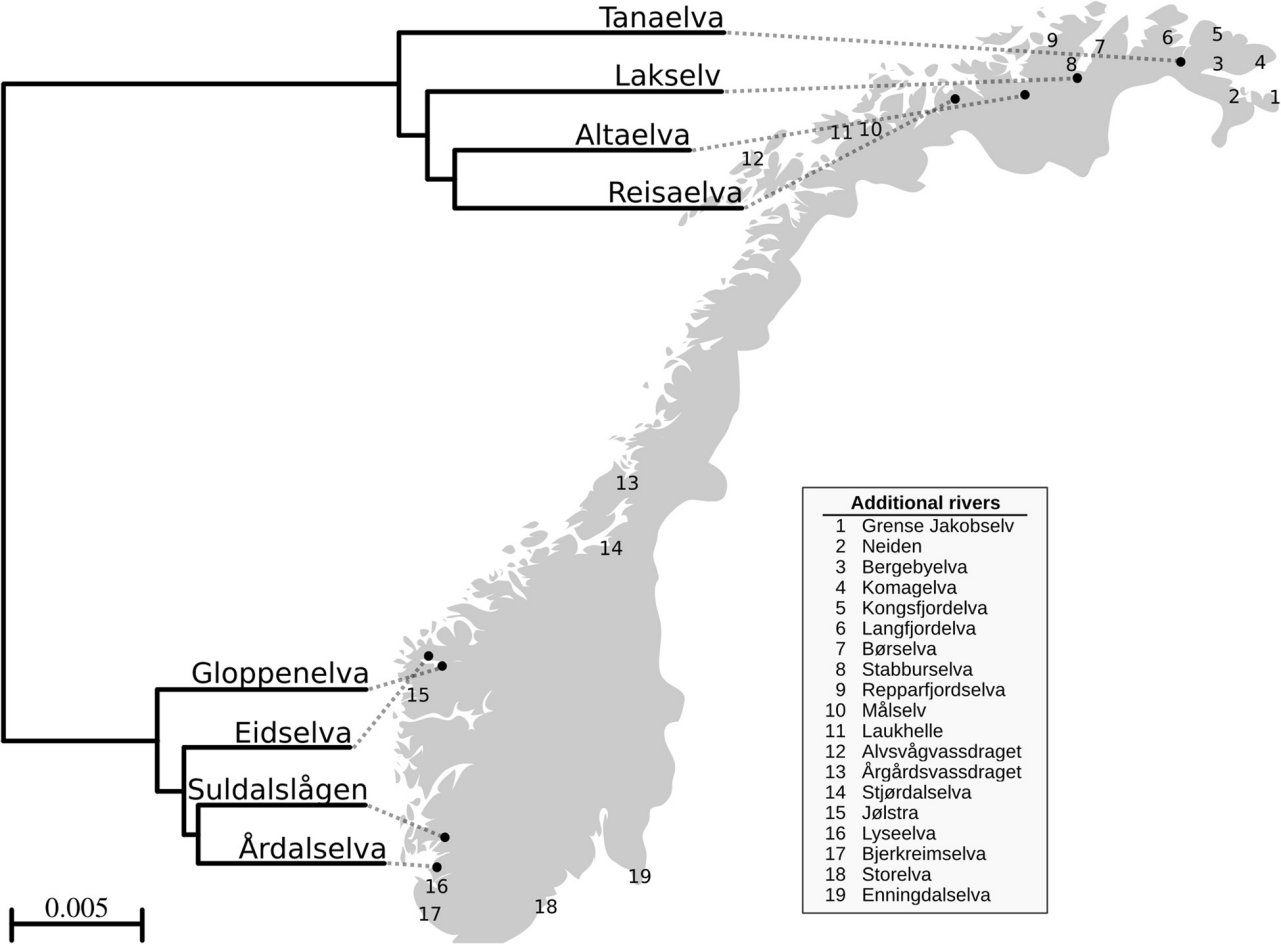
Geographical overview of sampled salmon populations. Resequenced genomes of Atlantic salmon from four populations in Northern Norway and four populations in Southern Norway were analyzed in this study and are shown as black dots on a map. A phylogenetic tree based on pairwise calculations of fixation index (F_ST_) illustrates the genetic distances between the sequenced populations. 19 additional populations from rivers along the Norwegian coast were analyzed using genotyping assays and the geographical sampling locations of these are indicated according to numbers in the map

Recently, a genome wide association study (GWAS) based upon whole genome resequencing data revealed a selective sweep in Atlantic salmon strongly associated with age of maturation [27]. Using a similar methodological approach, the present study aimed to identify genes and genomic regions diverging between Atlantic salmon populations in the north and south of Norway. In order to achieve this objective, salmon populations inhabiting the four rivers Tanaelva, Lakselv, Altaelva and Reisaelva from Northern Norway and the four rivers Gloppenelva, Eidselva, Suldalslågen and Årdalselva from Southern Norway were chosen for resequencing using DNA pools (*n* = 30 fish per river, Fig. 1). The major finding in this study was the observation that diversifying natural selection has acted on immune related genes causing adaptive divergence between populations in the north and south of Norway.

## Results and discussion

**Fig. 2:**
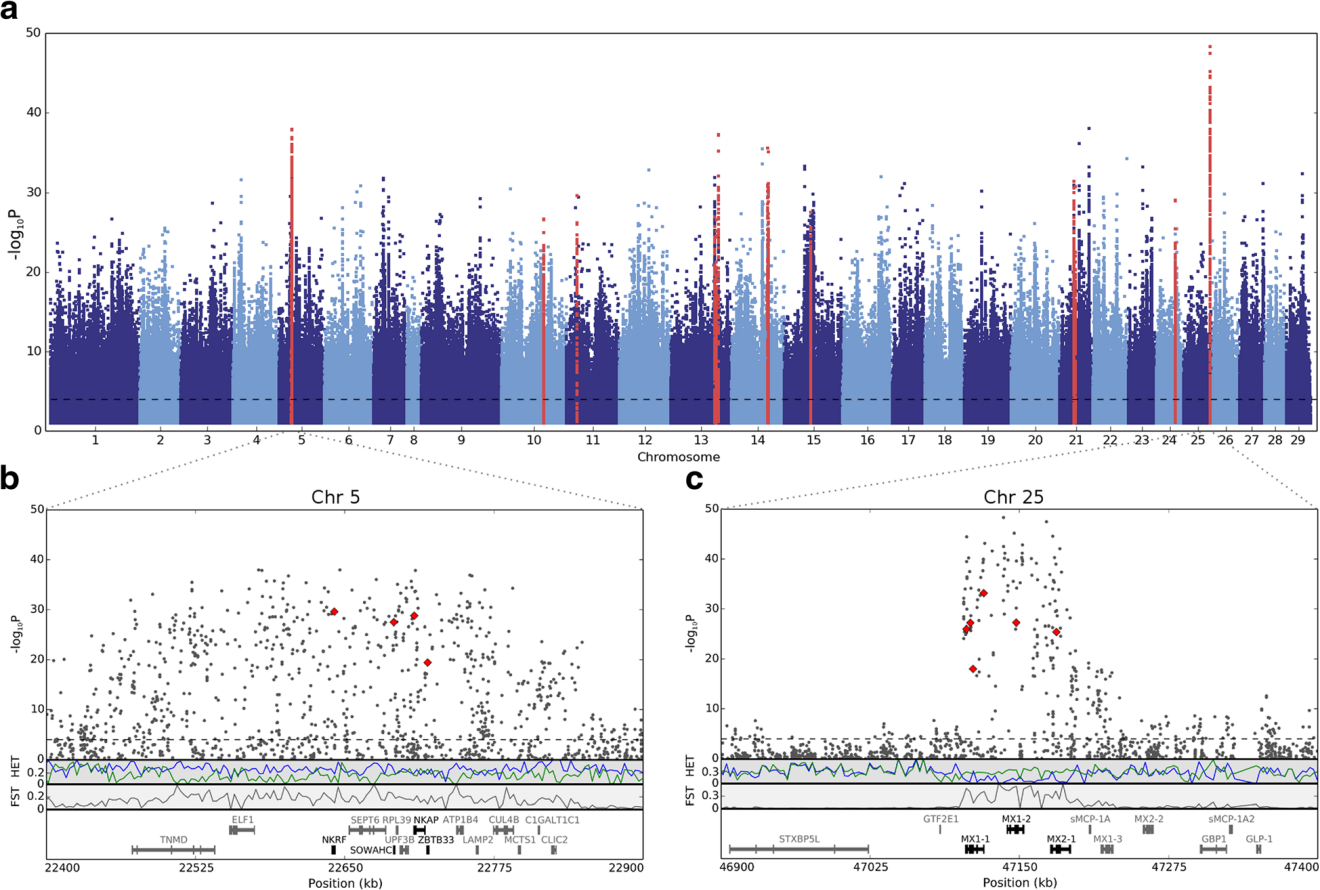
Identification of genomic regions under selection in Northern and Southern Norway. **a** Manhattan plot showing differentiated SNPs between northern and southern populations of Atlantic salmon in Norway. The x-axis indicates chromosomal positions; the y-axis presents the negative logarithm of the P-value for allele frequencies being different between the two geographical regions. SNPs in selective sweep regions identified using FST/heterozygosity are indicated by red dots. SNPs above the dashed horizontal line (*p* < 1.0658e-4, 0.1 % FDR) have significantly different allele frequencies between the two geographical regions. **b** and **c** Magnification showing 500 kb of selective sweeps on Chr 5 and Chr 25. SNPs are indicated as black dots and missense mutations are marked with red squares. The track labeled “HET” shows the heterozygosity of salmon from Northern (blue) and Southern Norway (green) in 3 kb windows. The track labeled “FST” shows the F_ST_ between populations in Northern and Southern Norway in 3 kb windows. In the bottom, identified genes are shown, with genes containing differentiated missense mutations colored black. The x-axis shows the chromosomal positions given in kb

Whole genome sequence data from eight selected rivers along the Norwegian coast (Fig. 1) was mapped to the most recent Atlantic salmon reference genome (AKGD00000000.4). This yielded a 26.7× average depth of coverage of uniquely mapped reads per river. SNP calling revealed 4,450,990 high quality SNPs. To quantify the genetic difference between populations of the chosen rivers, Hudson’s estimator for Wrigth’s fixation index (F_ST_) [34] was calculated (Additional file 1: Table S1). A phylogenetic tree was made using this distance matrix to illustrate and confirm the reported large genetic difference between the northern and southern populations of Atlantic salmon in Norway (Fig. 1). Statistical analysis using the Cochran-Mantel-Haenszel test for different allele frequencies between northern and southern Atlantic salmon populations revealed 474,410 SNPs with significantly different allele frequencies (0.1 % FDR, Fig. 2a). Genomic regions subjected to recent positive selection are expected to have lower heterozygosity than other regions, and if the selective pressure differs between populations, higher F_ST_ is observed [35]. An approach calculating F_ST_ and heterozygosity in 50 kb sliding windows has previously been used to identify genomic regions under selection (selective sweeps) [36]. This method was used to find selective sweeps which differ between northern and southern salmon populations in Norway (Additional file 1: Figure S1). The combined F_ST_/heterozygosity approach suggested 10 selective sweeps that differed between the two geographical regions. The sweeps ranged from 75,000 to 575,000 bp in size, and were found in chromosomes 5, 10, 11, 13–15, 21, 24 and 25 (Table 1). These sweeps contained in total 59 genes involved in a number of different biological processes including cell division, cytokinesis, angiogenesis, development, transcriptional regulation and immune response. For a detailed list of gene ID and short description of function see Additional file 1: Table S2.

**Table 1:** Selective sweeps. F_ST_ and heterozygosity estimations in sliding windows were used to identify differentiated loci undergoing selection in either northern or southern populations of Atlantic salmon in Norway. Genes carrying differentiated missense mutations are shown in bold

Chromosome	Chromosomal region	Sweep length (bp)	Genes in selective sweeps
5	22,475,000 – 22,800,000	325,000	*tnmd, elf1,* ***nkrf*** *, sept6,* ***sowahc*** *, rpl39, upf3b,* ***nkap*** *,* ***zbtb33*** *, atp1b4, lamp2, cul4b, mcts1, c1galt1c1, clic2*
10	76,250,000 – 76,450,000	200,000	*sept7,* ***anln*** *, prmt7, coq9, polr2c, dgat1, nedd4, adat1, spire2*
11	19,225,000 – 19,300,000	75,000	*numa1*
13	78,325,000 – 78,425,000	100,000	***trpc2*** *,* ***rrm1*** *, slc6a7,* ***rb1*** *, lpar6*
13	81,025,000 – 81,150,000	125,000	*eda2r, ar, ophn1*
14	64,700,000 – 65,275,000	575,000	*pard6g, bloc1s4, nutf2,* ***adnp2*** *, txnl4a, pqlc1, kcng2, ctdp1, adck5,* ***cpsf1*** *,* ***parp10*** *, st3gal1, khdrbs3*
15	46,925,000 – 47,200,000	275,000	*mdga1*
21	24,850,000 – 25,075,000	225,000	*trim13,* ***rnaseh2b*** *, nr0b1, il1rapl1*
24	34,225,000 – 34,525,000	300,000	*edil3*
25	47,075,000 – 47,225,000	150,000	*stxbp5l, gtf2e1,* ***mx1-1*** *,* ***mx1-2*** *,* ***mx2-1*** *, smcp-1a, mx1-3*

The high number of SNPs and genes in the selective sweeps complicates the task of pin-pointing the most important genetic differences. Therefore, we focused on missense mutations that induce amino acid changes in proteins, since these are more likely to confer a difference in biological function. Within the identified sweeps 20 significantly differentiated missense SNPs were found, comprising 15 different genes dispersed in 6 selective sweeps (Table 2). Three missense mutations were observed in the sweep on Chr 10, all in a single gene, *anln*, encoding an actin-binding protein required for cytokinesis. Three genes on Chr 13 harbor missense mutations: *trpc2*, involved in chemosensory transduction, and interestingly knockout mice display changes in their sexual, aggressive, and parenting behaviors [37]; *rrm1*, an enzyme essential for the production of deoxyribonucleotides; *rb1*, which promotes G0-G1 transition when phosphorylated by CDK3/cyclin-C acts as a transcriptional repressor of E2F1 target genes. Also in the Chr 14 sweep there are three genes with missense mutations: *adnp*, a homeodomain containing DNA binding transcription factor; *cpsf1*, encoding a component of the cleavage and polyadenylation specificity factor complex; *parp10*, encoding a ADP-ribosyltransferase involved in apoptosis, NF-kB signaling, and DNA damage repair [38]. The sweep on Chr 21 contains one gene, *rnaseh2b,* which is linked to a chronic inflammatory disorder in humans [39].

**Table 2:** Missense mutations in selective sweeps. Several missense mutations were discovered in the selective sweeps. The table lists resequencing derived reference alleles frequencies in the northern (N) and southern (S) populations of salmon in Norway. SNPs from Chr 5 and Chr 25 selected for genotyping are shown in bold

Chr	Position	-log_10_P	Reference allele frequency (S/N)	Ref/alt nucleotide	Ref/alt amino acid	Gene	Description of gene
**5** ^**a**^	**22,641,277**	**29.6**	**1.00/0.34**	**A (G)**	**Phe (Ser)**	***nkrf***	NF-kB repressing factor.
5	22,691,092	27.5	0.95/0.29	G (T)	Ala (Ser)	*sowahc*	Unknown function.
**5** ^**a**^	**22,708,279**	**28.8**	**1.00/0.33**	**C (T)**	**Val (Met)**	***nkap***	NF-kB activating protein.
**5** ^**a**^	**22,719,453**	**19.4**	**0.79/0.27**	**T (A)**	**Val (Glu)**	***zbtb33***	Transcriptional regulator.
10^b^	76,272,006	18.7	0.94/0.46	T (G)	Asp (Glu)	*anln*	Actin binding protein.
10	76,272,245	18.4	0.96/0.49	C (A)	Pro (Gln)	*anln*	Same as above.
10	76,278,414	14.5	0.32/0.76	G (A)	Ala (Thr)	*anln*	Same as above.
13	78,341,469	24.1	0.97/0.43	G (A)	Gly (Arg)	*trpc2*	Transient Receptor Cation Channel.
13	78,349,376	20.3	0.98/0.40	T (G)	Glu (Ala)	*rrm1*	Ribonucleoside-diphosphate reductase.
13^b^	78,413,379	20.7	0.98/0.48	A (T)	Gln (Leu)	*rb1*	Regulator of entry into cell division.
14	64,739,123	20.0	0.55/0.01	T (C)	Asn (Ile)	*adnp2*	Transcription factor.
14^b^	64,988,859	11.9	0.36/0.79	A (T)	Asn (Lys)	*cpsf1*	Pre-mRNAs processing.
14^b^	65,006,543	20.6	0.38/0.94	A (C)	Asp (Glu)	*parp10*	ADP-Ribosyltransferase.
21^b^	24,974,056	19.7	0.98/0.46	T (A)	Met (Lys)	*rnaseh2b*	Non catalytic subunit of RNase H2.
**25** ^**a**^	**47,105,598**	**26.0**	**0.84/0.03**	**A (G)**	**Thr (Ala)**	***mx1-1***	Interferon-induced antiviral.
25	47,108,912	27.2	0.91/0.00	G (A)	Val (Ile)	*mx1-1*	Same as above.
25	47,111,137	18.0	0.79/0.06	G (A)	Val (Met)	*mx1-1*	Same as above.
**25** ^**a**^	**47,120,121**	**33.1**	**0.83/0.06**	**C (T)**	**Arg (Cys)**	***mx1-1***	Same as above.
25	47,147,348	27.2	0.22/0.98	G (T)	Pro (His)	*mx1-2*	Same as above.
25	47,181,001	25.4	0.85/0.00	T (C)	His (Arg)	*mx2-1*	Same as above.

^a^Allele frequencies from genotyping are illustrated in Fig. 4

^b^Allele frequencies from genotyping are illustrated in Additional file 1: Figure S5

The second most significant selective sweep was found on Chr 5 (Fig. 2b) and included the stress and immune response transcription factor genes *nkrf* and *nkap*; *zbtb33* encoding a transcriptional regulator binding to methylated CpG dinucleotides, and a gene with unknown function, *sowahc*. Both Nkrf and Nkap are transcription factors which regulate the NF-kB pathway in which Nkap activates many cell processes including inflammation, immunity, differentiation, cell growth and apoptosis, while Nkrf mediates transcriptional repression of certain Nkap responsive genes. Since NF-kB signaling pathways activate the immune system in the host, these proteins are key targets for proteases expressed by invading pathogens [40]. Functional studies of the Nkap protein have revealed roles for this protein in T-cell maturation [41] and mRNA splicing [42]. To our knowledge, no previous studies have identified functionally significant SNPs associated with any of the four genes located within this sweep, however one of the SNPs found in *nkap* is located in a highly conserved region necessary for transcriptional repression. Here the valine is conserved in other species representing the ancestral variant while in Northern Norway methionine is most common (Additional file 1: Figure S2). This finding may be related to differences in immune defense between salmon from these two regions, a suggestion supported by the fact that the NF-kB pathway is differently regulated in IPN resistant salmon [43]. Further studies will reveal how these SNPs modulate the function of NF-kB and virus response or if other functional properties are associated with the selective sweep on Chr 5.

The most significant sweep was found on Chr 25 and contained a cluster of five *mx* (myxovirus resistance) genes known to be involved in defense against viruses. Three of these *mx* genes contained missense mutations; *mx1-1*, *mx1-2* and *mx2-1* (Fig. 2c). These proteins are dynamin-like GTPases induced upon virus infection through the innate interferon system. It has been shown that they can act broadly against both DNA and RNA viruses and specifically against certain viruses [44] and studies in mouse, human and chicken have shown that single missense mutations in *Mx1* and *Mx2* can confer such specific responses [45–48]. It is possible that the identified missense SNPs in the *mx* genes reflect specific adjustments to different viral disease pressures between northern and southern populations of salmon. We identified missense SNPs in all regions of the protein including a SNP in the antiviral specificity domain in exon 13 (Additional file 1: Figure S3). This SNP represents a structurally relevant amino acid substitution, where arginine seems to be the ancestral variant and cysteine the derived variant dominating in the northern population (Chr 25 position: 47,120,121). Likewise, SNPs in this domain have been associated with specific virus resistance in chicken [49, 50] and pig [51]. SNPs in *mx* genes have also been investigated in another fish species, the turbot [52], however, properties related to protection against viruses were not investigated in this study. In rainbow trout (*Oncorhynchus mykiss*) genetic variation in *mx* between strains in exon 3–6, was correlated with susceptibility to infectious hematopoietic necrosis virus (IHNV) [53]. This virus also infects Atlantic salmon and our discovery of a missense mutation in exon 6 suggests that salmon could have adapted to the IHNV (Additional file 1: Figure S3). In addition, different strains of rainbow trout display variable susceptibility to this virus [54]. In this study we cannot elucidate the functional significance of the acquired SNPs in *mx* in Northern Norway, however, further studies will reveal whether any of these changes have been involved in host-virus adaptation [55].

**Fig. 3:**
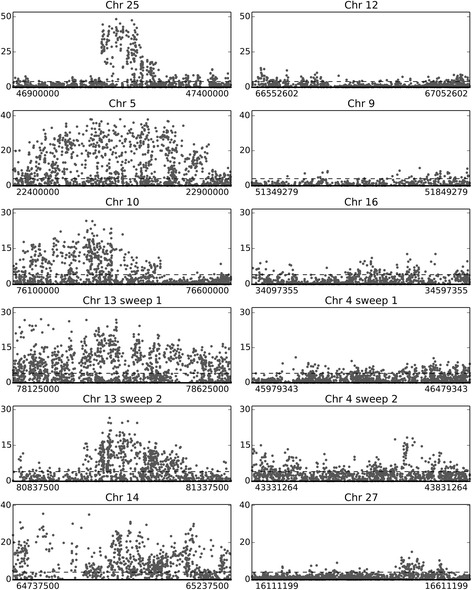
Comparison of sweeps and paralogous regions. Representation of the selective sweeps having paralogous regions in the salmon genome, displaying a 500 kb overview of the SNPs in the selective sweeps (left side) and in the corresponding paralogous regions (right side). The y-axis shows –log_10_P of the SNPs being different in populations between the north and south of Norway, and the x-axis represents the position in the given chromosome. The dashed lines indicate the genome wide significance threshold (0.1 % FDR)

We also investigated whether the selective sweeps on Chr 5 and Chr 25 had paralogous regions in the partially tetraploid salmon genome [56]. *In silico* analysis showed that both sweeps have paralogous regions located on other chromosomes. The Chr 5 sweep has a paralogous region on Chr 9 (Additional file 1: Figure S4, position 51,349,279 to 51,849,279), which did not contain any differentiated SNPs. The synteny is conserved in other species, and the existence of only one copy in zebrafish (*Danio rerio*), combined with the observation that missense mutations on Chr 5 are not present in the paralogous genes on Chr 9, indicate that the mutations arose after the salmonid specific whole genome duplication (WGD). Based upon this observation, it is possible to speculate that the WGD provided paralogous regions where one copy was free to sub- or neo-functionalize, much like the theory for duplicated genes [57] which has been suggested to be important for evolutionary adaptation and innovation in salmon [58], in teleosts [59] and in general [30]. A similar picture is seen for the sweep on Chr 25 where the paralogous region harbors a cluster of three *mx* genes on Chr 12 (position 66,552,602 to 67,052,602), but carries no differentiated SNPs or missense mutations. While the sweeps on Chrs 11, 15, 21 and 24 have no clear paralogous regions, the sweeps on Chr 10, Chr 14 and the two sweeps on Chr 13 also have paralogous regions with very few significantly differentiated SNPs, on Chr 16, 27 and 4, respectively (Fig. 3). Similarly, in our recent discovery of the loci in Chr 25 controlling age at maturity [27] we investigated the two paralogous regions in Chr 21, both of which were without SNPs associated with the trait. Together, these findings indicate that the partially tetraploid stage may be beneficial for adaptation, since one gene copy or gene cluster can keep the original function while the other can adapt to a new situation such as novel disease pressures.

**Fig. 4:**
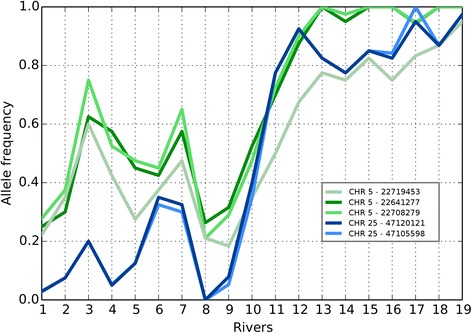
Allele frequencies of five missense mutations in populations along the Norwegian coast. Three missense mutations identified in selective sweeps on Chr 5 and two on Chr 25 were used in a genotyping assay of Atlantic salmon populations along the Norwegian coast. The graphs show frequencies of the reference alleles. River numbers are explained in Fig. 1. SNPs on Chr 5 included missense mutations in the genes *nkrf*, *nkap* and *zbtb33* (green lines) and missense mutations on Chr 25 were located in the *mx1-1* gene (blue lines)

In this study, the initial resequencing was based only upon males. This is because it allowed reusing sequence data from our previous work [27]. The targeted SNP analysis, used to validate the results from resequencing in a larger independent set of rivers, was conducted using both males and females (Figs. 1 and 4). Genotyping of mixed sex salmon from 19 rivers (*n* = 20 salmon/river) along the Norwegian coast (Fig. 1) for five missense SNPs on Chr 5 and 25 confirmed strong genetic differentiation between salmon populations from the north and south of Norway (Fig. 4). Populations from northern rivers (1–9) displayed allele frequencies in the range 0–0.7, while those from southern rivers (11–19) were close to fixation for one allele at these two loci. Salmon from river 10, Målselv, shows intermediate frequencies, which corresponds well with what has been reported in the literature [31, 32]. These results also confirm allele frequency estimations from the pooled resequencing (Table 2). In addition, we designed Sequenom assays for five other missense SNPs in other regions; one SNP each for sweeps on Chrs 10, 13 and 21, and two SNPs in Chr 14. Genotyping was performed for all 19 rivers (Additional file 1: Figure S5). The allele frequencies showed the same clear difference between the northern and southern populations. For the SNPs on Chr 14 there appears to be an additional genetic shift between the rivers 14, Stjørdalselva and rivers south of this. In addition to the data produced within the present study, resequencing data from a recent publication was downloaded and compared to our results [28]. The downloaded data include three individually sequenced salmon from 4 southern and 3 northern salmon rivers in Norway. These data corroborate our resequencing and genotyping results (Additional file 1: Table S3). Our surveyed SNPs therefore also represent robust and good genetic markers for distinguishing northern and southern populations of Atlantic salmon in Norway. Future studies on an extended set of populations may reveal if these are also robust markers for detecting genetic structuring in other parts of the distribution range of the species.

Atlantic salmon aquaculture involves rearing domesticated fish that originate from commercial breeding programs. Forty wild populations from both the north and south of Norway were sampled when establishing the national breeding programs for salmon [60]. However, analyses of genetic markers demonstrate that there is a dominance of salmon from Southern Norway in the domesticated lines currently in production [61]. Genetic analyses of farmed salmon escapees in Norway have uncovered genetic introgression into native salmon populations in both Northern and Southern Norway, but the biological consequence remains unknown [32, 61, 62]. Consequently the results from the present study, where adaptive genetic divergence between wild salmon from populations located in the north and south of Norway was revealed, it is likely that the potential negative genetic impact of domesticated salmon introgression is greater in populations located in northern regions, since the farmed fish originate mostly from wild Southern Norway populations.

## Conclusion

In this study we performed a GWAS by genome resequencing with the aim to screen the Atlantic salmon genome for genetic differentiation between the northern and southern populations in Norway. By investigating eight rivers we uncovered ten particularly striking sweeps including two clusters of immune related genes harboring missense mutations. A feasible interpretation is that different populations of Atlantic salmon have historically been exposed to different selection pressures in the form of pathogens. Some of these adapted alleles could be advantageous for aquaculture production which is currently hampered by a number of diseases, including virus infections [63]. Future studies should include gene editing of immune genes found in these selective sweeps [64, 65] in combination with viral exposure experiments. Within these experiments, viruses relevant to salmon aquaculture should be the primary focus since finding specific resistance alleles can be of significant value to the industry and could also be used for protecting wild fish against high disease pressures posed by open cage aquaculture [66]. Upon finding the protective alleles, selective breeding on individuals with beneficial haplotypes could lead to increased welfare for aquaculture salmon, decreased disease pressure on wild populations and could also be economically favorable for the industry. On the other hand, further studies should investigate the impact of genetic introgression from fertile aquaculture escapees on the adaptive genetic properties in wild populations. To reduce the risk of this unwanted loss of local adaptation and alteration of fitness-related traits, a sustainable solution would be the use of sterile fish in aquaculture, especially in Northern Norway. Future studies should also investigate whether paralogous regions of selective sweeps have undergone positive selection or not, as the latter scenario would suggest an evolutionary mechanism which provides higher adaptive possibilities when a genome is partially tetraploid.

## Methods

### Samples and sampling

Scales from 30 Atlantic salmon males per river were selected from a sample set of 26,000 samples collected in coastal fisheries in Northern Norway. In the Kolarctic Salmon project (http://prosjekt.fylkesmannen.no/Kolarcticsalmon), the multilocus genotypes of all individuals were compared to a genetic baseline consisting of over 180 rivers from Northern Russia and Norway and were assigned to river of origin. Samples that were assigned with high probability to four rivers in Northern Norway; Altaelva, Reisaelva, Lakselv and Tanaelva were generously made available to this study. 30 salmon males from each of four different rivers in Southern Norway, including Årdalselva, Eidselva, Gloppenelva and Suldalslågen were sampled and resequenced in a recent study [27]. In addition to these, we also used male and female salmon DNA from 19 rivers along the Norwegian coast. These included 20 parr individuals from each of the rivers Grense Jakobselv, Neiden, Bergebyelva, Komagelva, Kongsfjordelva, Langfjordelva, Børselva, Stabburselva, Repparfjordselva, Målselv, Laukhelle, Alvsvågvassdraget, Årgårdsvassdraget, Stjørdalselva, Jølstra, Lyseelva, Bjerkreimselva, Storelva and Enningdalselva (represented by numbers in Fig. 1). With the exception of Enningdalselva where the sample was obtained from scales collected by recreational fisheries, these samples were obtained from fins collected by electrofishing of juvenile salmon from mulitple locations in the rivers.

### DNA extraction and sequencing

DNA from the 19 rivers for genotyping was extracted from scales or fin samples using Qiagen DNeasy Blood and Tissue Kit (Qiagen, Hilden, Germany) according to manufacturer’s recommendations. From salmon belonging to the four populations in Northern Norway total DNA was extracted from scales using Qiagen DNeasy Blood and Tissue Kit. Equal amounts of DNA from ten individuals were pooled to make three pools per river, totaling 30 individuals from each river. Paired-end libraries were constructed using the Genomic DNA Sample Preparation Kit (Illumina, CA, USA) according to manufacturer’s instructions and sequenced on the Illumina HiSeq2000 platform (Illumina, CA, USA) at the Norwegian Sequencing center (https://www.sequencing.uio.no, Oslo, Norway) with each pool sequenced in separate lanes.

### Sequence mapping and SNP calling

To ensure high quality sequences, sequenced reads were inspected with FastQC (http://www.bioinformatics.babraham.ac.uk/projects/fastqc/). Adapter sequence removal and quality trimming was done with Cutadapt [67], resulting in 1,077,839,448 (SD 40,407,492) paired reads on average per river. Sequenced reads were mapped to the most recent release of the salmon genome (AGKD0000000.4) using Bowtie2 (v.2.1.0) [68] without soft clipping (end-to-end mode). To increase the sensitivity of the mapping, seed length (−L parameter) was set to 18 and the interval between extracted seeds (−i parameter) was set to S,1,1.5 corresponding to the function f (L) = 1 + 1.5*sqrt (L), where L is the read length. Additionally, the maximum number of mismatches per seed (−N parameter) was set to L,0,0.1, corresponding to the function f (L) = 0 + 0.1*L, where L is the read length, and minimum alignment score (−−score-min parameter) was set to L,-0.6,-0.4, corresponding to the function f (L) = −0.6 + −0.4*L, where L is the read length. To remove ambiguously mapped reads the mapping quality threshold was set to 20. To obtain higher sequence coverage, the three sequenced pools per river were merged to a single BAM file using SAMtools merge. SNPs were called using SAMtools mpileup [69] and the output was parsed using the PoPoolation2 package (mpileup2sync.jar) [70] with a minimum base quality threshold of 20. For a SNP to be included in the final set of high quality SNPs, minimum coverage of 10 and maximum coverage of 50 (99 % percentile) was required for each river. In addition, the total number of observed minor alleles was required to be at least 8. Recently published whole genome resequencing data from individuals [28] was downloaded and mapped to the reference genome. The data included three salmon from each of the rivers Tanaelva, Repparfjordelva, Altaelva, Namsenelva, Årgårdsvassdraget, Nausta and Jølstra, where the first three represent populations in Norhtern Norway and the last four represent Southern Norway. Accession numbers for the samples are shown in the caption of Additional file 1: Table S3.

### Statistical analysis

Pairwise fixation index (F_ST_) between all eight sequenced populations was calculated for all high quality SNPs using Hudson’s estimator for F_ST_ [34]. F_ST_ values were averaged over all SNPs in each population to generate a distance matrix using F_ST_ as genetic distance. This matrix was converted to a newick tree using NEIGHBOR from the Phylip package [71] and a phylogenetic tree was created with NJplot [72]. To find SNPs with significantly different allele frequencies (0.1 % FDR) between populations from Northern and Southern Norway the Cochran-Mantel-Haenszel test for repeated tests of independence from the PoPoolation2 package (cmh-test.pl) [70] was used. The FDR threshold was determined using the method described in [73]. Allele counts for each river were merged to get the total allele count per SNP in Northern and Southern Norway, corresponding to 120 individuals per geographical region. From this, F_ST_ values between the northern and southern populations were estimated using the F_ST_ calculation from the PoPoolation2 package (fst-sliding.pl) for each SNP, with --pool-size parameter set to 120. Genomic regions with low values of heterozygosity may indicate SNPs under selection. Therefore heterozygosity values were estimated for north and south of Norway, separately, for each SNPs as 2 * (major allele frequency * minor allele frequency). Sliding windows of 50 kb with steps of 25 kb was used to find genomic regions with high F_ST_ values and with low heterozygosity values in either Northern or Southern Norway. This approach is similar to one used to discover genomic regions under selection in other animals [36]. To identify putative selective sweeps it was required that the average F_ST_ value of the window was at least 0.17 (above 99.9 % percentile) and that average heterozygosity of the window in either Northern or Southern Norway was at most 0.15 (below 5 % percentile) (Additional file 1: Figure S1). The thresholds were chosen with focus on capturing the outliers in the FST and heterozygosity distributions. Putative sweeps were extended to the sides for as long as the neighboring windows had either average F_ST_ of at least 0.17 or heterozygosity of at most 0.15 in either Northern or Southern Norway. If identified sweeps were less than 50 kb apart these were joined to avoid fragmentation of the putative selective sweeps. Genomic windows containing more than 10 % ambiguous bases (Ns) in the reference assembly were discarded to exclude regions with high levels of uncertainty.

### SNP annotation

Genes in the sweep regions were obtained from the official genome annotation (NCBI Salmo salar Annotation Release 100). Missense mutations in selective sweep regions were identified by manual inspection of the coding sequences. Amino acid sequences of five *mx* genes found in a selective sweep on Chr 25 were aligned to the homologs Mx1 and Mx2 from human and MxD and MxG from Zebrafish using BLASTP (default parameters). Functional domains in the Mx proteins were assigned using domain information for human Mx1 from UniProt. Amino acid sequences from four genes containing missense mutations in a selective sweep on Chr 5 (*nkrf*, *sowahc*, *nkap* and *zbtb33*) were aligned to homologous zebrafish and Northern Pike genes using BLASTP with default parameters. Synteny between genes in the sweep on Chr 5 and other animals was found using the UCSC genome browser (https://genome.ucsc.edu) to inspect the syntenic regions of zebrafish, human and mouse. Paralogous regions of the sweeps were identified using TBLASTN (default parameters) with the genes in the sweeps against the salmon genome.

### Genotyping

Twenty salmon from 19 rivers along the Norwegian coastline (*n* = 380) were genotyped using ten of the most significant missense mutations on a Sequenom MassARRAY iPLEX platform (San Diego, CA, USA). Primers and extension primers are listed in Additional file 1: Table S4. The genotyping primers were designed to not target any paralogous genes in the genome.

## Abbreviations

bp, base pai; Chr, chromosome; FDR, false discovery rate; FST, fixation index; GWAS, genome-wide association study; IHNV, infectious hematopoietic necrosis virus; IPN, infectious pancreatic necrosis; kb, kilo bases; SNP, single nucleotide polymorphism; WGD, whole genome duplication

## Additional file

Additional file 1: Tables S1 to S4, Figs S1 to S5. Supplemental Materials. (DOC 2321 kb)
