# Supplementary material for: Atlantic salmon populations reveal adaptive divergence of immune related genes - a duplicated genome under selection
Source: BMC Genomics. 2016 Aug 11;17:610. doi: 10.1186/s12864-016-2867-z (PMC4982270; doi:10.1186/s12864-016-2867-z)
Supplement: Additional file 1: Tables S1 to S4, Figs S1 to S5. — Supplemental Materials. (DOC 2321 kb) [file 12864_2016_2867_MOESM1_ESM.doc]

**Supplemental Material for**

**Atlantic Salmon Populations Reveal Adaptive Divergence of Immune Related Genes - A duplicated genome under selection**

Erik Kjærner-Semb^1,2*^, Fernando Ayllon^1^, Tomasz Furmanek^1^, Vidar Wennevik^1^, Geir Dahle^1^, Eero Niemelä^3^, Mikhail Ozerov^4^, Juha-Pekka Vähä^4,6^, Kevin A Glover^1,2^, Carl J Rubin^5^, Anna Wargelius^1^, Rolf B Edvardsen^1*^

^1^ Institute of Marine Research, Bergen, Norway

^2^ Department of Biology, University of Bergen, Norway

^3^ Natural Resources Institute Finland, Helsinki, Finland

^4^ Kevo Subarctic Research Institute, University of Turku, Turku, Finland

^5^ Department of Medical Biochemistry and Microbiology, Uppsala University, Uppsala, Sweden

^6^Association for Water and Environment of Western Uusimaa, Finland

*Corresponding authors

E-mail: erikkj@imr.no, rolfbe@imr.no

**This file includes**

Supplemental Tables S1 to S4

Supplemental Figs. S1 to S5

**Supplemental Table S1. Pairwise F_ST_ between sequenced populations**. Hudson’s estimator for F_ST_ was used to calculate pairwise F_ST_ between all eight pool-sequenced populations of Atlantic salmon from Norway using the set of high quality SNPs.

|  | **Årdalselva** | **Eidselva** | **Gloppenelva** | **Suldalslågen** | **Altaelva** | **Lakselv** | **Reisaelva** |
| --- | --- | --- | --- | --- | --- | --- | --- |
| **Eidselva** | 0.0279 |  |  |  |  |  |  |
| **Gloppenelva** | 0.0333 | 0.0309 |  |  |  |  |  |
| **Suldalslågen** | 0.0272 | 0.0267 | 0.0322 |  |  |  |  |
| **Altaelva** | 0.0805 | 0.0773 | 0.0788 | 0.0780 |  |  |  |
| **Lakselv** | 0.0812 | 0.0779 | 0.0791 | 0.0785 | 0.0402 |  |  |
| **Reisaelva** | 0.0826 | 0.0794 | 0.0810 | 0.0801 | 0.0401 | 0.0493 |  |
| **Tanaelva** | 0.0818 | 0.0785 | 0.0798 | 0.0792 | 0.0441 | 0.0518 | 0.0502 |

**Supplemental Table S2. Extended information on genes in selective sweeps.** This table lists the genes, including gene ids, of genes located in the ten selective sweeps identified in this study.

| **Chr** | **Sweep** | **Gene** | **Genbank ID** | **Gene ID** | **Description** |
| --- | --- | --- | --- | --- | --- |
| 5 | 22,475,000 – 22,800,000 | *tnmd* | XM_014199076.1 | 106604450 | Angiogenesis |
| 5 | 22,475,000 – 22,800,000 | *elf1* | XM_014199069.1 | 106604448 | Immune response, transcription factor |
| 5 | 22,475,000 – 22,800,000 | *nkrf* | XM_014199066.1 | 106604447 | Transcriptional repressor for NFKB |
| 5 | 22,475,000 – 22,800,000 | *sept6* | XM_014199062.1 | 106604445 | Cytokinesis |
| 5 | 22,475,000 – 22,800,000 | *sowahc* | XM_014199059.1 | 106604443 | Unknown function |
| 5 | 22,475,000 – 22,800,000 | *rpl39* | NM_001141015.1 | 100195986 | Ribosomal protein |
| 5 | 22,475,000 – 22,800,000 | *upf3b* | XM_014199058.1 | 106604442 | mRNA processing |
| 5 | 22,475,000 – 22,800,000 | *nkap* | XM_014199056.1 | 106604441 | Transcriptional repressor |
| 5 | 22,475,000 – 22,800,000 | *zbtb33* | XM_014199055.1 | 106604440 | Transcriptional regulator |
| 5 | 22,475,000 – 22,800,000 | *atp1b4* | XM_014199047.1 | 106604437 | Transcriptional regulator |
| 5 | 22,475,000 – 22,800,000 | *lamp2* | XM_014199045.1 | 106604436 | Lysosome related |
| 5 | 22,475,000 – 22,800,000 | *cul4b* | XM_014199043.1 | 106604435 | DNA repair |
| 5^1^ | 22,475,000 – 22,800,000 | *mcts1* | Pos 22795499 - 22797344 | | Translation |
| 5 | 22,475,000 – 22,800,000 | *c1galt1c1* | XM_014199041.1 | 106604434 | Protein folding |
| 5 | 22,475,000 – 22,800,000 | *clic2* | NM_001140560.1 | 100195531 | Chloride channel protein |
| 10 | 76,250,000 – 76,450,000 | *sept7* | XM_014124354.1 | 106560914 | Cytokinesis |
| 10 | 76,250,000 – 76,450,000 | *anln* | XM_014124340.1 | 106560912 | Cytokinesis |
| 10 | 76,250,000 – 76,450,000 | *prmt7* | XM_014124417.1 | 106560946 | Methylation |
| 10 | 76,250,000 – 76,450,000 | *coq9* | XM_014124339.1 | 106560911 | Lipid binding |
| 10 | 76,250,000 – 76,450,000 | *polr2c* | NM_001141873.1 | 100196851 | Transcription |
| 10 | 76,250,000 – 76,450,000 | *dgat1* | XM_014124333.1 | 106560908 | Lipid synthesis |
| 10 | 76,250,000 – 76,450,000 | *nedd4* | XM_014124324.1 | 106560906 | Ubiquitin binding |
| 10 | 76,250,000 – 76,450,000 | *adat1* | XM_014124321.1 | 106560904 | RNA processing |
| 10 | 76,250,000 – 76,450,000 | *spire2* | XM_014124319.1 | 106560903 | Intracellular transport |
| 11 | 19,225,000 – 19,300,000 | *numa1* | XM_014126645.1 | 106562084 | Cell division |
| 13 | 78,325,000 – 78,425,000 | *trpc2* | XM_014137691.1 | 106567864 | Cation channel |
| 13 | 78,325,000 – 78,425,000 | *rrm1* | XM_014137690.1 | 106567863 | Cell division |
| 13^1^ | 78,325,000 – 78,425,000 | *slc6a7* | Pos 78378062 - 78379385 | | Transporter protein |
| 13^1^ | 78,325,000 – 78,425,000 | *rb1* | Pos 78384837-78416346 | | Cell division |
| 13 | 78,325,000 – 78,425,000 | *lpar6* | XM_014137689.1 | 106567862 | Signal transduction |
| 13^1^ | 81,025,000 – 81,150,000 | *eda2r* | Pos 81029006 - 81032747 | | Receptor |
| 13 | 81,025,000 – 81,150,000 | *ar* | XM_014137515.1 | 106567785 | Receptor, development |
| 13 | 81,025,000 – 81,150,000 | *ophn1* | XM_014137514.1 | 106567784 | Signal transduction |
| 14 | 64,700,000 – 65,275,000 | *pard6g* | XM_014142331.1 | 106570232 | Cell division |
| 14 | 64,700,000 – 65,275,000 | *bloc1s4* | XM_014142330.1 | 106570231 | Lysosome related |
| 14 | 64,700,000 – 65,275,000 | *nutf2* | XM_014142329.1 | 106570230 | Transporter protein |
| 14 | 64,700,000 – 65,275,000 | *adnp2* | XM_014142328.1 | 106570229 | Transcriptional regulation |
| 14 | 64,700,000 – 65,275,000 | *txnl4a* | XM_014142326.1 | 106570228 | RNA processing |
| 14 | 64,700,000 – 65,275,000 | *pqlc1* | XM_014142321.1 | 106570225 | Membrane protein |
| 14 | 64,700,000 – 65,275,000 | *kcng2* | XM_014142320.1 | 106570224 | Potassium channel protein |
| 14 | 64,700,000 – 65,275,000 | *ctdp1* | XM_014142316.1 | 106570222 | Cell division |
| 14 | 64,700,000 – 65,275,000 | *adck5* | XM_014142318.1 | 106570223 | Kinase |
| 14 | 64,700,000 – 65,275,000 | *cpsf1* | XM_014142314.1 | 106570221 | RNA processing |
| 14 | 64,700,000 – 65,275,000 | *parp10* | NM_001140613.1 | 100195584 | Histone modification |
| 14 | 64,700,000 – 65,275,000 | *st3gal1* | XM_014142310.1 | 106570220 | Glycosylation |
| 14 | 64,700,000 – 65,275,000 | *khdrbs3* | XR_001320628.1 | 106570219 | RNA processing |
| 15 | 46,925,000 – 47,200,000 | *mdga1* | XM_014144811.1 | 106571582 | Neuron migration |
| 21 | 24,850,000 – 25,075,000 | *trim13* | XM_014164604.1 | 100194870 | Ubiquitin binding |
| 21^1^ | 24,850,000 – 25,075,000 | *rnaseh2b* | Pos 24971693 - 24976605 | | Transcription |
| 21 | 24,850,000 – 25,075,000 | *nr0b1* | XM_014164619.1 | 106581998 | Embryonic development |
| 21 | 24,850,000 – 25,075,000 | *il1rapl1* | XM_014164642.1 | 106582011 | Neural system |
| 24 | 34,225,000 – 34,525,000 | *edil3* | XM_014172354.1 | 106585765 | Angiogenesis and embryonic development |
| 25 | 47,075,000 – 47,225,000 | *stxbp5l* | XM_014174624.1 | 106586892 | Intracellular transport |
| 25^1^ | 47,075,000 – 47,225,000 | *gtf2e1* | Pos 47090178 - 47090287 | | Transcription factor |
| 25 | 47,075,000 – 47,225,000 | *mx1-1* | XM_014174614.1 | 106586887 | Immune response |
| 25 | 47,075,000 – 47,225,000 | *mx1-2* | XM_014174616.1 | 106586889 | Immune response |
| 25 | 47,075,000 – 47,225,000 | *mx2-1* | XM_014174617.1 | 106586890 | Immune response |
| 25 | 47,075,000 – 47,225,000 | *smcp-1a* | XM_014174606.1 | 106586880 | Sperm motility |
| 25 | 47,075,000 – 47,225,000 | *mx1-3* | XM_014174615.1 | 106586888 | Immune response |
| 25^2^ | 47,075,000 – 47,225,000 | *mx2-2* | XM_014174618.1 | 106586891 | Immune response |

^1^ Gene not found in reference annotation, but was found using BLAST against Swissprot. The start and end positions are shown instead of gene ID.

^2^ *mx2-2* was included for completeness.

**Supplemental Table S3. Missense SNPs in resequenced individuals.** The table presents number of individuals in each genotype class for each missense SNP using three individually resequenced salmon from seven populations in Norway. Numbers are presented as number of homozygous for the reference allele / number of heterozygotes / number of homozygous for the alternative allele. Total numbers for north and south are shown in bold. Accession numbers for the samples are: SAMEA3533094, SAMEA3533095, SAMEA3533096, SAMEA3533109, SAMEA3533110, SAMEA3533111, SAMEA3533101, SAMEA3533100, SAMEA3533102, SAMEA3533107, SAMEA3533108, SAMEA3533106, SAMEA3533104, SAMEA3533103, SAMEA3533105, SAMEA3533097, SAMEA3533098, SAMEA3533099, SAMEA3533112, SAMEA3533118 and SAMEA3533120.

| **Chr** | **Pos** | **Tanaelva^1^** | | **Repparfjordelva^2^** | **Alta^1^** | **North total** | **Namsen** | **Årgårdsvassdraget^2^** | **Nausta** | **Jølstra^2^** | **South total** |
| --- | --- | --- | --- | --- | --- | --- | --- | --- | --- | --- | --- |
| 5 | 22,641,277 | 0/2/1 | 0/1/2 | | 2/0/1 | **2/3/4** | 3/0/0 | 3/0/0 | 2/1/0 | 3/0/0 | **11/1/0** |
| 5 | 22,691,092 | 0/1/2 | 0/2/1 | | 0/2/1 | **0/5/4** | 2/1/0 | 3/0/0 | 0/3/0 | 3/0/0 | **8/4/0** |
| 5 | 22,708,279 | 0/1/2 | 0/1/2 | | 0/1/1 | **0/3/5** | 3/0/0 | 3/0/0 | 2/1/0 | 3/0/0 | **11/1/0** |
| 5 | 22,719,453 | 0/1/2 | 0/1/2 | | 0/1/1 | **0/3/5** | 3/0/0 | 3/0/0 | 1/1/0 | 3/0/0 | **10/1/0** |
| 10 | 76,272,006 | 0/1/1 | 1/1/0 | | 0/0/0 | **1/2/1** | 3/0/0 | 3/0/0 | 3/0/0 | 2/1/0 | **11/1/0** |
| 10 | 76,272,245 | 0/2/1 | 1/2/0 | | 0/2/0 | **1/6/1** | 3/0/0 | 3/0/0 | 2/0/0 | 2/1/0 | **10/1/0** |
| 10 | 76,278,414 | 2/1/0 | 2/0/1 | | 1/2/0 | **5/3/1** | 0/2/1 | 1/0/2 | 1/1/1 | 1/1/1 | **3/4/5** |
| 13 | 78,341,469 | 2/1/0 | 1/2/0 | | 1/0/2 | **4/3/2** | 3/0/0 | 1/2/0 | 3/0/0 | 3/0/0 | **10/2/0** |
| 13 | 78,349,376 | 2/0/0 | 1/2/0 | | 1/0/2 | **4/2/2** | 2/0/0 | 1/2/0 | 2/1/0 | 3/0/0 | **8/3/0** |
| 13 | 78,413,379 | 2/0/1 | 1/2/0 | | 1/0/1 | **4/2/2** | 3/0/0 | 1/2/0 | 2/1/0 | 3/0/0 | **9/3/0** |
| 14 | 64,739,123 | 0/0/1 | 0/0/3 | | 0/0/2 | **0/0/6** | 0/3/0 | 2/0/1 | 0/2/0 | 0/1/2 | **2/6/3** |
| 14 | 64,988,859 | 0/1/0 | 3/0/0 | | 1/1/0 | **4/2/0** | 0/2/1 | 1/1/1 | 0/1/2 | 0/2/1 | **1/6/5** |
| 14 | 65,006,543 | 2/1/0 | 3/0/0 | | 3/0/0 | **8/1/0** | 0/3/0 | 2/0/1 | 0/1/2 | 0/2/1 | **2/6/4** |
| 21 | 24,974,056 | 1/1/1 | 1/0/1 | | 0/2/0 | **2/3/2** | 2/0/0 | 3/0/0 | 3/0/0 | 3/0/0 | **11/0/0** |
| 25 | 47,105,598 | 0/0/3 | 0/0/3 | | 0/0/2 | **0/0/8** | 1/2/0 | 2/1/0 | 2/1/0 | 3/0/0 | **8/4/0** |
| 25 | 47,108,912 | 0/0/2 | 0/0/3 | | 1/0/0 | **1/0/5** | 1/2/0 | 2/1/0 | 3/0/0 | 3/0/0 | **9/3/0** |
| 25 | 47,111,137 | 0/0/3 | 0/0/3 | | 3/0/0 | **3/0/6** | 1/2/0 | 2/1/0 | 3/0/0 | 3/0/0 | **9/3/0** |
| 25 | 47,120,121 | 0/0/3 | 0/0/3 | | 0/0/3 | **0/0/9** | 1/2/0 | 2/1/0 | 1/1/0 | 3/0/0 | **7/4/0** |
| 25 | 47,147,348 | 3/0/0 | 3/0/0 | | 2/0/0 | **8/0/0** | 0/3/0 | 0/1/2 | 0/0/3 | 0/1/2 | **0/5/7** |
| 25 | 47,181,001 | 2/1/0 | 0/0/3 | | 0/0/2 | **2/1/5** | 1/2/0 | 2/1/0 | 2/1/0 | 3/0/0 | **8/4/0** |

^1^ Population also represented in pool sequencing.

^2^ Population also represented in genotyping assays.

**Supplemental Table S4. Primers for genotyping assays.**

| **Chr** | **SNP position** | **Forward primer** | **Reverse primer** | **Un-extended primer (UEP)** |
| --- | --- | --- | --- | --- |
| 5 | 22719453 | ACCTGCTTCTTATGGATCCC | GCACTTTCATCCCCAAACTC | GATCCCTATTATCTCTGGGGTT |
| 5 | 22641277 | TCATGGAAGCCAAGGATCCG | ATGTGTCCGGATGCCTAGTG | ggggCTCGGCCAGATACGAGT |
| 5 | 22708279 | GAAGAGTAGTAATGTACCTGC | CTAGTGACGAAATCGCAACC | ttgggAATGTACCTGCTACTGCTCAT |
| 10 | 76272006 | CTAGTTGGGACTGAACTACC | AGATGTGGGTTCCCCTTTAG | CTGAACTACCTCCTCTTC |
| 13 | 78413379 | ATGAACTGATGAGAGACCGC | GCATGTCGAAATTGCATTTG | AGACCGCCACCTGGACC |
| 14 | 64988859 | TTGCTGGAGGCTTGTCTTTC | TTGCCATGACATGTGGACTG | GCTTGTCTTTCTTCTTGCC |
| 14 | 65006543 | CAGCCTTCAACATGATATCAG | CCGGGCCATATTAGTGTTTG | ACACAGACTGAATATATTCTTACC |
| 21 | 24974056 | TTGGTCTTGCTGTGTACCTG | CTCCCACTGAGATGTTAGTC | gcGGCTGAGAAGACAGTGA |
| 25 | 47120121 | GTATCGTGCAAAGTTGCAGG | GACATCAAACTCTCACAGGC | TTGCAGGCCCAATTGGAT |
| 25 | 47105598 | TTTCCTGAACTCTGGTCTCC | GACAACATGAGATCCATAGG | GTCTCCCACTACAGCAATGG |

**
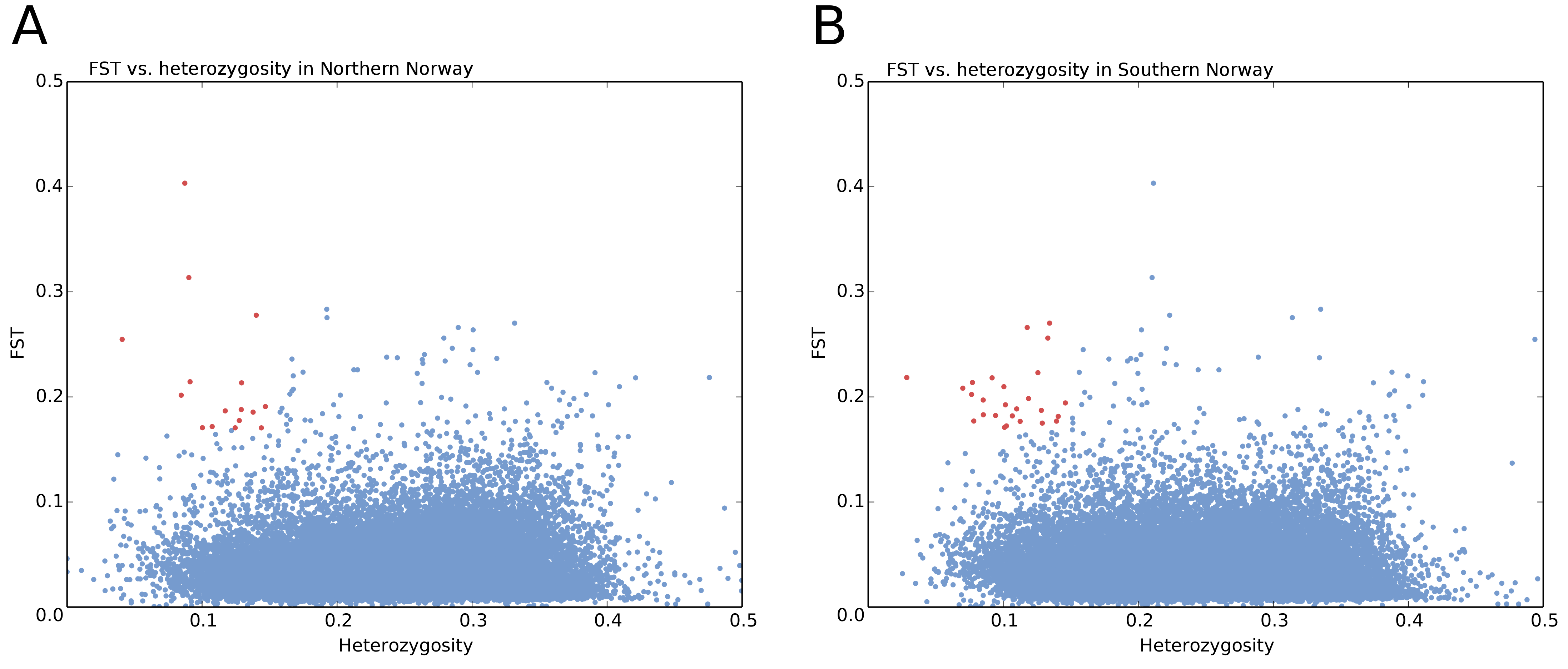
**

**Supplemental Figure S1. F_ST_ vs. heterozygosity for 50 kb windows.** F_ST_ and heterozygosity values were calculated for 50 kb windows for every 25 kb along the salmon genome. Red dots indicate windows that initiate selective sweep regions. (**A**) F_ST_ vs. heterozygosity in Northern Norway. (**B**) F_ST_ vs. heterozygosity in Southern Norway.

**
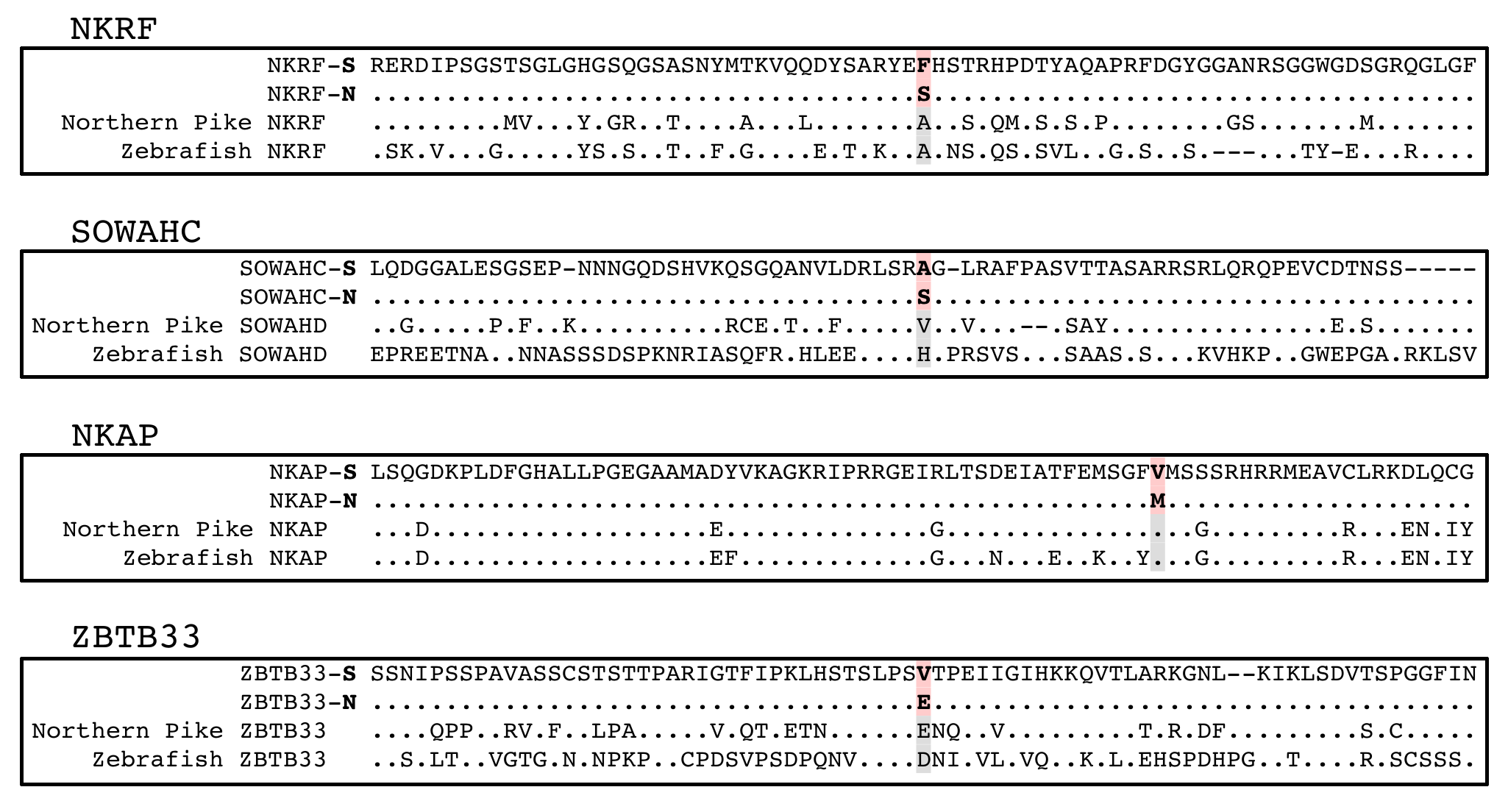
Supplemental Figure S2. Multiple sequence alignments of genes in Chr 5.** Four genes in the selective sweep on Chr 5 contained missense mutations; *nkrf*, *sowahc*, *nkap* and *zbtb33*, and are shown aligned to the Northern Pike and Zebrafish homologs. Amino acid differences between the northern and southern populations are shown in red. Dots indicate identical amino acid as in the Southern Norway salmon. Genbank IDs for Zebrafish: *nkrf*: NP_001004499.3; *sowahd*: XP_001339865.1; *nkap*: NP_001003414.1; *zbtb33*: NP_001093478.1. Genbank IDs for Northern Pike: *nkrf*: XP_012988866.1; *sowahd*: XP_010894486.1; *nkap*: XP_010894544.1; *zbtb33*: XP_010894556.1.


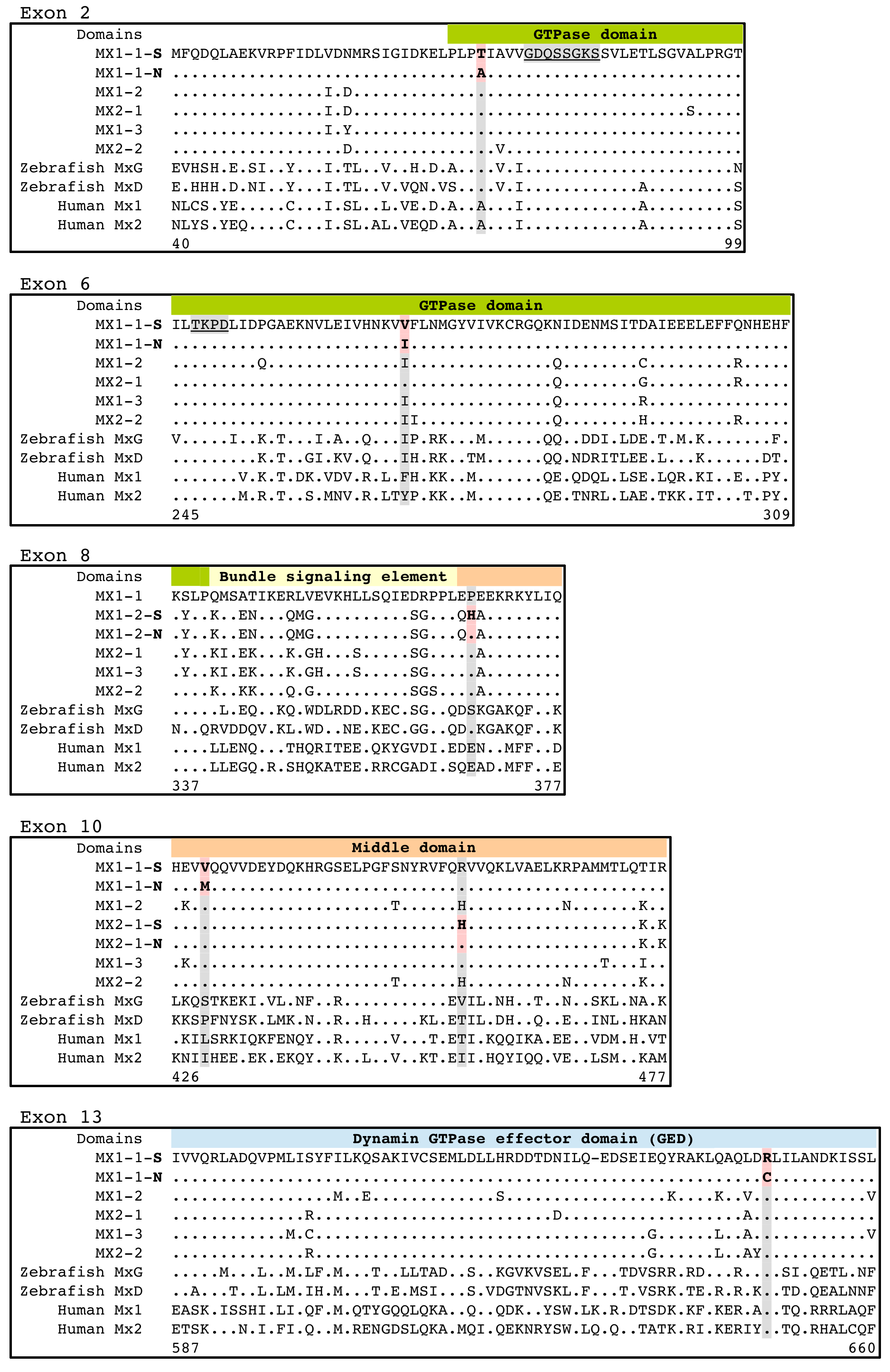


**Supplemental Figure S3. Multiple sequence alignment of *mx* genes.** Amino acid sequences of five *mx* genes from the selective sweep on Chr 25, two Zebrafish (Genbank ID MxG: NP_001116443.1; MxD: XP_695562.6) and two Human (Genbank ID Mx1: NP_001138397.1; Mx2: NP_002454.1) *Mx* genes were aligned. Only exons containing missense mutations are shown. Dots indicate identical amino acid as in *mx1-1*-S. Amino acid differences between northern and southern populations are colored red and are shown on separate lines with the gene name followed by either S (south) or N (north). GTP-binding domains are shown in grey with underline. Functional domains and amino acid positions corresponding to human Mx1 are shown above and below the sequences.


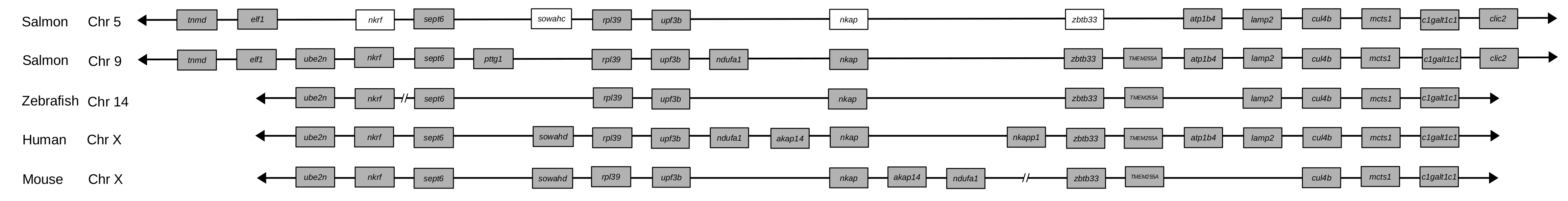


**Supplemental Figure S4. Genes in Chr 5 in synteny.** Genes found in the selective sweep on Chr 5 were found to be in synteny with other animals, as well as a region in Chr 9 of the salmon genome. The top line presents the genes in the selective sweep on Chr 5, with genes containing missense mutations in white. Double slash indicates genes interspanned by 5 - 25 other genes. Not to scale.


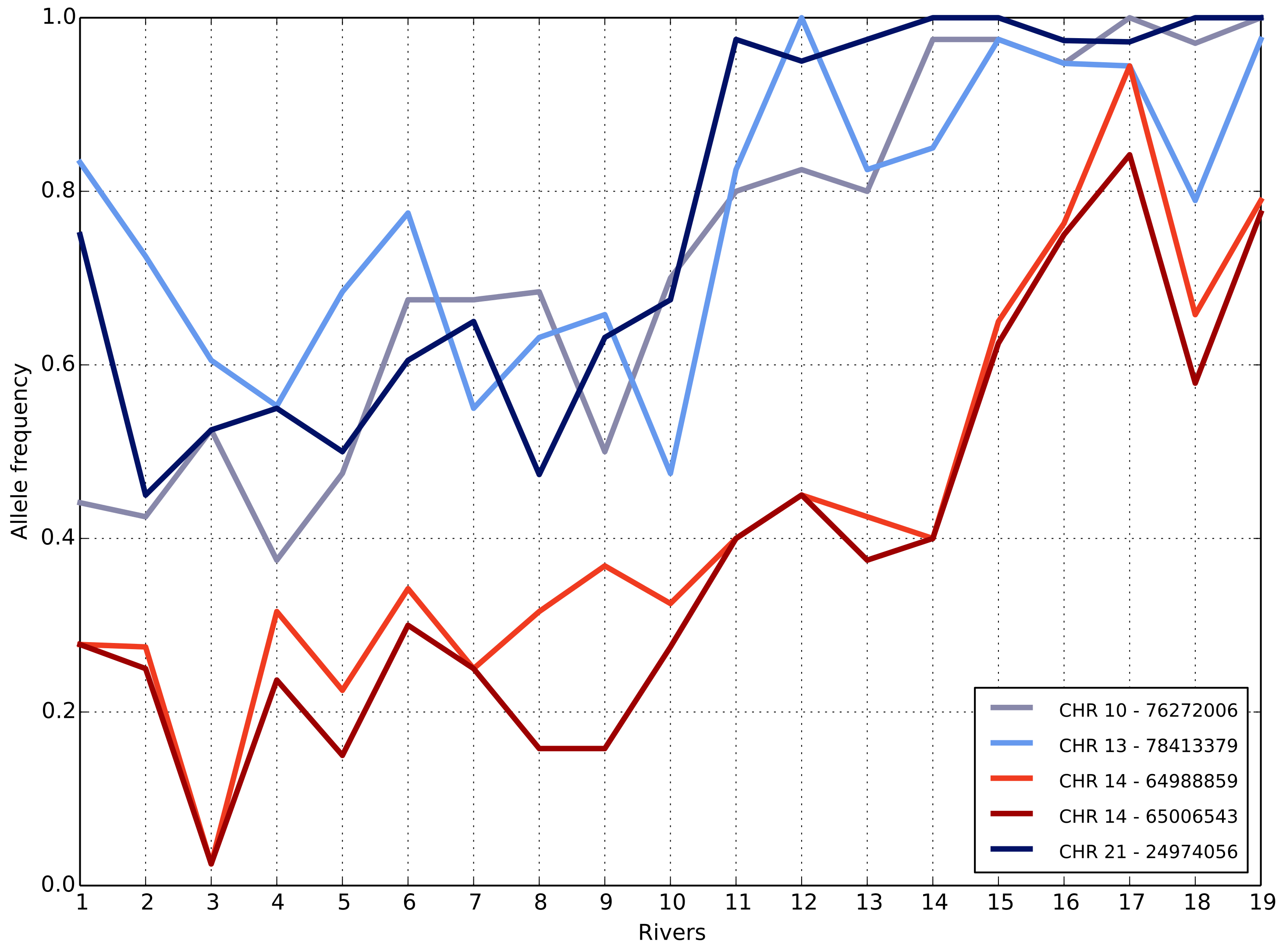
**Supplemental Figure S5. Allele frequencies of additional missense SNPs along the Norwegian coast.** Five missense mutations in addition to the five SNPs shown in Fig 4 were genotyped in populations along the Norwegian coast. The graphs show the frequencies of the reference alleles. Missense SNPs in selective sweeps on: Chr 10 (*anln*), Chr 13 (*rb1*), Chr 14 (*cpsf1* and *parp10*) and Chr 21 (*rnaseh2b*). River numbers are explained in Fig 1.
